# Using topology to tame the complex biochemistry of genetic networks

**DOI:** 10.1098/rsta.2011.0548

**Published:** 2013-02-13

**Authors:** Mukund Thattai

**Affiliations:** National Centre for Biological Sciences, Tata Institute of Fundamental Research, UAS/GKVK Campus, Bellary Road, Bangalore 560065, India

**Keywords:** synthetic biology, feedback loops, Boolean threshold models

## Abstract

Living cells are controlled by networks of interacting genes, proteins and biochemicals. Cells use the emergent collective dynamics of these networks to probe their surroundings, perform computations and generate appropriate responses. Here, we consider genetic networks, interacting sets of genes that regulate one another’s expression. It is possible to infer the interaction topology of genetic networks from high-throughput experimental measurements. However, such experiments rarely provide information on the detailed nature of each interaction. We show that topological approaches provide powerful means of dealing with the missing biochemical data. We first discuss the biochemical basis of gene regulation, and describe how genes can be connected into networks. We then show that, given weak constraints on the underlying biochemistry, topology alone determines the emergent properties of certain simple networks. Finally, we apply these approaches to the realistic example of quorum-sensing networks: chemical communication systems that coordinate the responses of bacterial populations.

## 1. Introduction

Genes are physically embodied as a string of nucleotide bases (ATGGCCCTG…) on a self-replicating DNA molecule, contained within the cytoplasm of a prokaryote or the nucleus of a eukaryote. Genes encode proteins, which in turn carry out the processes required for the maintenance of cellular life. During the process of gene expression, the genetic information is first transcribed or copied onto a short-lived messenger RNA (mRNA) molecule. This mRNA is then translated repeatedly into a protein, as specified by the genetic code: a set of three consecutive nucleotides of mRNA uniquely specifies one of twenty possible amino acids, a series of which are strung together to form the protein (the short sequence above, for example, encodes the first three amino acids of the human insulin protein).

**Figure 1.**
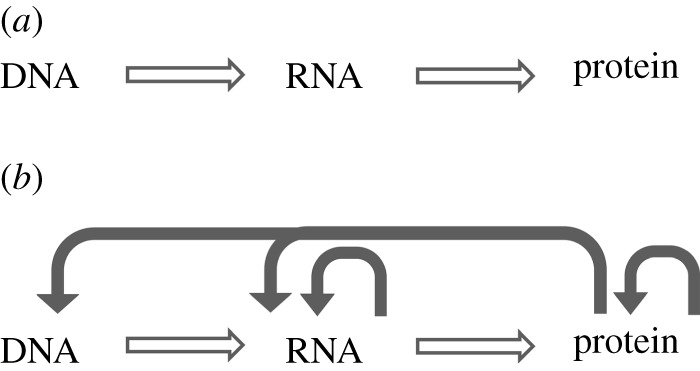
(*a*) The central dogma of molecular biology. (*b*) A more accurate representation of the central dogma, with filled arrows representing potential regulatory interactions.

This basic description, the ‘central dogma of molecular biology’ (figure 1*a*), is not the entire story however. Every cell in the human body carries the same complement of genes, yet a heart cell and a brain cell are made up of very different proteins. Even in a single-celled organism such as a bacterium, different proteins are expressed at different times. The bacterium *Escherichia coli* is able to assemble flagella when it needs to swim, and pili when it needs to anchor itself to a surface; it will produce a metabolic enzyme only when its substrate is present, and synthesize DNA repair proteins only when subject to shock. In short, genes can be turned on and off.

This simple but powerful idea was first proposed by Jacques Monod in the 1940s [1], and the framework he constructed remains essentially unchallenged to this day. The expression of genes is a tightly regulated process [2], ch. 7. Central to this process is a control element known as a promoter—a short stretch of DNA that precedes every gene. The promoter contains a binding site for the RNA polymerase, the protein complex responsible for transcription. Correspondingly, mRNAs contain binding sites for ribosomes, the protein complexes responsible for translation. The rate of transcription at a promoter can be increased or decreased by proteins known as transcription factors that bind DNA in the vicinity of the promoter. In prokaryotes, transcription factors typically bind within a few tens of bases of the promoter, whereas in eukaryotes, long-distance interactions between transcription factors and the RNA polymerase can extend over megabases. Eukaryotes also have additional ‘epigenetic’ mechanisms to regulate transcription, via covalent modifications of the histone proteins on which DNA is wrapped, or modifications of the DNA itself. Once an mRNA molecule is transcribed, its rate of translation can be regulated by proteins that influence the capacity of ribosomes to bind ribosome binding sites, or by protein complexes that degrade specific mRNAs. Additionally, it has become clear that a significant fraction of transcribed RNAs do not encode proteins; rather, many of these non-coding RNAs can themselves regulate the translation of mRNAs to proteins, via the sophisticated machinery of RNA interference [3]. Taking all these effects into account, the central dogma must be modified with a few additional arrows (figure 1*b*).

These new arrows are loaded with implications: they permit us to assemble complex networks of transcriptional and regulatory interactions. Gene *A* can activate gene *B* and gene *C*, but repress gene *D*, and so on. There is a compelling case to be made for the existence of such networks in living cells. Consider that a bacterial genome contains about 4000 genes, whereas the human genome contains about 25 000 genes—a surprisingly modest difference at first glance, given that the human body is made up of more than 200 cell types, not to mention higher degrees of organization required to specify a complex tissue such as the brain. A deeper analysis suggests that gene number is not the correct measure of complexity: the properties of a cell are specified by the proteins contained within it; the range of possible cell types is therefore determined by the range of possible *combinations* of expressed genes, and grows exponentially with gene number. How are all such combinations to be accessed, however? We know that distinct external signals can drive cells to differentiate into distinct types. However, such signals do not directly interact with individual genes, turning them on or off. Once the differentiation process is triggered, various combinations of gene expression must arise through the intrinsic behaviour of the genes themselves. That is, there must be a network of genetic interactions which, based on very few external regulatory cues, is able to produce the correct expression patterns. The manifest complexity of cellular behaviour strongly implies the existence of complex regulatory networks within.

In recent times, we have been able to resolve network architecture in unprecedented detail using high-throughput biochemical experiments, or by inference from gene expression and gene knockout data [4–8]. For certain well-studied organisms such as *Escherichia coli* and the yeast *Saccharomyces cerevisiae*, there is a growing body of detailed information regarding transcriptional and regulatory interactions [9–12]. When these data are combined, what emerges is a picture of highly structured networks with rich topologies [13], containing recurring motifs or patterns [14,15], very different from randomly connected sets of genes. Just as individual proteins have been selected for function, entire networks seem to be similarly selected. So here is what one might call the central idea of network biology: that the complex behaviour of living cells must be understood as emerging not just from the properties of individual genes, but from the manner in which they are connected.

## 2. The control of gene expression

**Figure 2.**
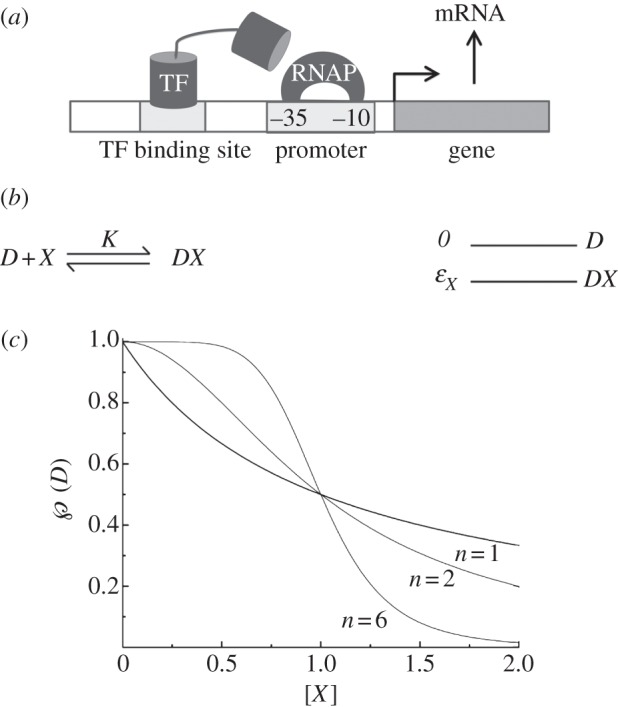
Protein–DNA interactions. (*a*) Genes are DNA regions that are transcribed into mRNA, and eventually translated into proteins. Promoters are DNA regions upstream of genes where the RNA polymerase molecule (RNAP) binds and initiates transcription. Transcription factors (TFs) can bind near promoters and interact with the polymerase, exerting regulatory control. (*b*) The binding of a single protein is shown in the reaction-kinetic (left) and energetic (right) representations. (*c*) Free DNA as a function of protein levels. The graphs are of Hill functions, showing hyperbolic (*n*=1) as well as sigmoidal (*n*=2, *n*=6) binding curves. Higher Hill coefficients produce more threshold-like functions. The half-saturation concentration is one in each case.

For the purposes of this exposition, we focus on prokaryotic gene regulation via promoters. A promoter is a loosely defined object. We can take it to signify a stretch of DNA, upstream of every gene, which controls whether that gene is expressed or not. The properties of a promoter, like those of a gene, are determined by its DNA sequence. A survey of bacterial promoters reveals a conserved pattern of nucleotides, all variations of a particular consensus sequence. The most conserved regions are two short stretches situated −35 and −10 nucleotides from the site at which transcription begins [2, ch. 7]. These regions are thought to provide the binding site that is specifically recognized by the RNA polymerase protein (figure 2*a*).

There are in fact numerous proteins that, like the polymerase, are able to recognize and bind specific nucleotide sequences. Their binding sites are typically between six and 20 base pairs in length. Binding is mediated by physical interactions between residues on the protein and on the DNA molecule. Given the structure of a protein we should, in principle, be able to calculate its interaction energy with a particular DNA sequence. The result of such a calculation would be the ‘DNA-binding code’. The search for such a code is an active area of research [16–18], but for the time being we can rely on experimental measurements of binding affinities [7,8]. Various classes of DNA-binding proteins are known, grouped according to the structure of their DNA recognition domains. These proteins are often modular, having one domain that binds DNA, and another that is responsible for regulatory interactions. Once bound to DNA, a protein can recruit other proteins to its vicinity, or can prevent them from binding. In particular, a DNA-binding protein can interact with and influence the binding and transcriptional activity of the RNA polymerase. Such molecules are known as gene regulatory proteins or transcription factors. They can be classified as activators (which increase the rate of polymerase binding) or repressors (which prevent the polymerase from binding or block it from transcribing). A given protein might activate or repress transcription depending on the relative position of its binding sequence to that of the RNA polymerase.

The activity of a transcription factor can itself be modulated by the binding of small molecules or by covalent modification [2], chs 7 and 15. For example, the *E. coli lac* repressor, which blocks transcription at the lac operon, contains binding sites for a sugar called allolactose; when the repressor is bound to allolactose, it is unable to bind DNA, and therefore unable to repress transcription. This type of modulation is a key mechanism by which external signals can regulate gene expression. Many small molecules in the environment can diffuse across the bacterial cell membrane to directly influence intracellular transcription factors. Other types of signalling molecules can bind the extracellular domains of transmembrane proteins known as receptors; this causes a conformational change in the receptor’s intracellular domain, which can drive the subsequent activation or inhibition of transcription factors by phosphorylation. For example, a large number of bacterial ‘two-component systems’, consisting of a membrane-bound sensor and intracellular transcriptional regulator, operate on this principle. As we show later, these types of regulatory inputs influence intracellular network dynamics, allowing cells to sense environmental conditions and respond appropriately.

We can calculate the expression level at a particular promoter from a biophysical model that incorporates the microscopic details just mentioned, using an approach pioneered by Shea & Ackers [19] in their study of the O_R_ control system of bacteriophage *λ*. To do this, we first list all possible promoter configurations (the combinations in which the promoter binds various regulatory proteins or the RNA polymerase); and we specify the relative free energies of each of these states. Once this information is given, there is a well-defined thermodynamic prescription for calculating system properties. Consider a DNA region D that can bind a set of proteins *Xi* (*i*=1,…,*n*), each with multiplicity *m*_*i*_. Let the cytoplasmic protein concentrations be [*Xi*]. This binding event can be represented as
2.1
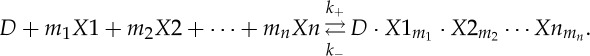

For simplicity in the discussion that follows, this representation clubs together what are in fact several independent binding events, and includes effective rate constants *k*_+_ and *k*_−_ for this clubbed reaction. Indeed, there might be several configurations of the bound state: other combinations in which the DNA can bind these proteins. Let *s*_*j*_ represent these various states (including the one in which the DNA is bare). The probability of occurrence of each state in thermodynamic equilibrium is then [19]
2.2
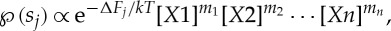

where *k* is Boltzmann’s constant, and *T* is the absolute temperature. The term *ΔF* is the standard free energy of the given configuration, describing the energetics of interaction between the molecules; for example, bonds between DNA and protein residues can stabilize binding by making *ΔF* more negative. The concentration terms arise owing to entropy or counting: the higher the concentration of a certain protein, the more ways in which one can pick a single molecule to bind the DNA.

We can give this result a kinetic interpretation, under the simplifying assumption of a clubbed multi-protein reaction. The probability that *m*_1_ molecules of *X*1 enter the reaction volume will be proportional to [*X*1]^*m*_1_^. More generally, the probability per unit time that the reaction (2.1) occurs from left to right (*P*_+_) or right to left (*P*_−_) is
2.3
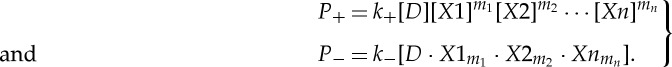

If these were the only possible reactions, then in equilibrium we would have *P*_+_=*P*_−_, giving
2.4
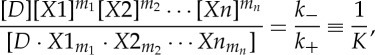

where *K* is the equilibrium constant. This result is usually presented as the principle of mass action. The concentration of a given promoter state is the total DNA concentration multiplied by the probability of occurrence of that state. If we agree to measure all free energies as differences from that of the bare configuration, a comparison of (2.2) and (2.4) shows
2.5
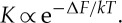

That is, the values of the reaction rate constants are constrained by free-energy differences: their ratio must be consistent with the equilibrium prediction. There is in fact a much more basic constraint on the kinetic constants. Imagine that the DNA is involved in several complexes. In that case the condition *P*_+_=*P*_−_, while sufficient to ensure time-invariance of probabilities, is certainly not necessary. It could be that the depletion of a certain species through one reaction is compensated for, not by the reverse reaction, but by a separate creation pathway. However, detailed balance asserts that in equilibrium such solutions are not acceptable: all forward reactions must be balanced by the corresponding reverse reactions. This fact is not at all evident from a reaction-kinetic formulation. While it will be convenient to work within the kinetic framework of rate constants, we must always bear in mind the constraints imposed by equilibrium considerations.

We can now use these general results to study a few relevant examples, where we now explicitly treat multi-step reactions. Consider a DNA region *D* to which the protein *X* can bind. For convenience, let us measure energy in units of *kT*, and let the free energy of the bare DNA be zero. Suppose the free energy of state *DX* is *ε*_*X*_ (figure 2*b*). The probability that the DNA is bare is given by
2.6
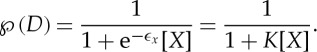

The concentration of bare DNA is a hyperbolic function of the protein concentration, reaching half-saturation at a value [*X*]=1/*K* (figure 2*c*).

**Figure 3.**
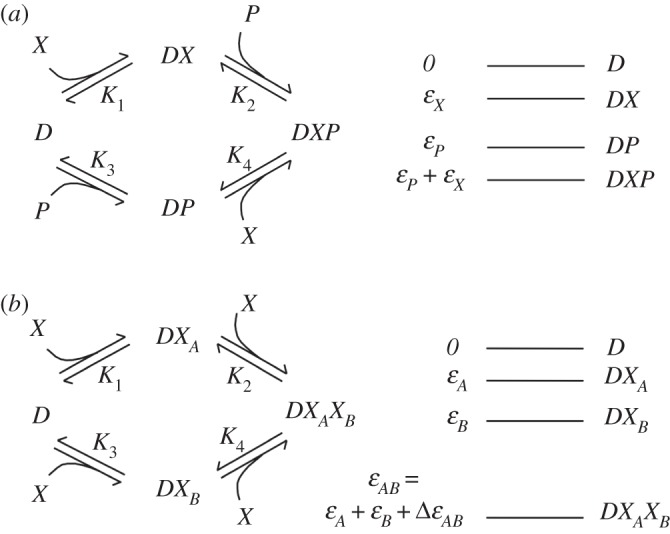
Transcriptional regulation by DNA-binding proteins. (*a*) Independent binding of a repressor (*X*) and the polymerase (*P*). The free energy of the doubly bound state is the sum of the individual binding energies. (*b*) Cooperative binding. The binding of a single molecule of *X* increases the likelihood that a second molecule will bind.

Suppose now that the DNA region *D* represents a promoter, and that the protein *X* is a repressor, which acts to prevent transcription by the polymerase *P*. Let the free energy of the state *DP* be *ε*_*P*_. If the two proteins *X* and *P* bind independently, then the free energy of the doubly bound state *DXP* will be the sum of the individual binding energies. (If the independent-binding assumption is not valid, the energy of the state *DXP* must be provided as an additional parameter.) The energies of the various bound states in this scenario are indicated in figure 3*a*. The only state from which transcription can proceed is the state *DP*. Applying the equilibrium prescription, we find that this state occurs with probability
2.7
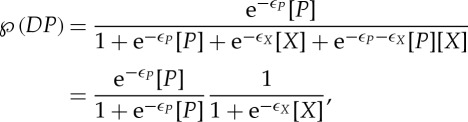

where we have explicitly factorized the expression. This factorization is possible precisely because the proteins *X* and *P* bind independently, so the probability that state *DP* occurs is the probability that *P* is bound multiplied by the probability that *X* is not bound (the latter being given by (2.6)). It is instructive to see how the derivation might proceed from the kinetic framework. Applying detailed balance, we can find two expressions for the concentration of the doubly bound state, corresponding to the upper and lower binding paths:
2.8
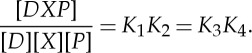

The four dissociation constants cannot, therefore, be independently specified. (Note also that, by the independent binding property, *K*_1_=*K*_4_=*e*^−*ε*_*X*_^ and *K*_2_=*K*_3_=*e*^−*ε*_*P*_^.)

In many instances, transcription factors bind to multiple sites. Suppose the promoter in question contains two sites, *A* and *B*, to which *X* can bind in any order (figure 3*b*). Let the free energies of the two singly bound states be *ε*_*A*_ and *ε*_*B*_, and that of the doubly bound state be *ε*_*AB*_=*ε*_*A*_+*ε*_*B*_+*Δε*_*AB*_. These assumptions correspond to the most general situation, of which the following are special cases: if the two sites are identical, then *ε*_*A*_=*ε*_*B*_; if *X* binds independently to these sites, then *Δε*_*AB*_=0. The energy term *Δε*_*AB*_ corresponds to some interaction between the two bound copies of *X*. If the binding of a single molecule makes it more favourable for another to bind, a condition referred to as positive cooperativity, then *Δε*_*AB*_<0. Conversely, in a situation of negative cooperativity, *Δε*_*AB*_>0, and the binding of one molecule interferes with the ability of the other to bind. Positive cooperativity is the norm among transcription factors that act multiply. Let us see what effect this will have. Assume, for simplicity, that |*ε*_*A*_|∼|*ε*_*B*_|≪|*Δε*_*AB*_|. In the kinetic framework, this corresponds to *K*_1_≪*K*_2_, and *K*_3_≪*K*_4_, with the detailed balance condition again as shown in (2.8). We find
2.9
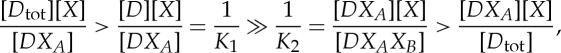

where the inequalities are obtained by noticing that the concentration of any DNA configuration must be less than that of the total amount of DNA available. This shows that [*DX*_*A*_]≪[*D*_tot_], and similarly, [*DX*_*B*_]≪[*D*_tot_]: the singly bound configurations form a negligible fraction of the population. No sooner has one molecule of *X* bound DNA, than the second also binds. Therefore, the probability that the DNA is bare is given by
2.10
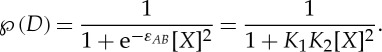

The cooperativity of binding gives rise to the quadratic term in the denominator. The binding curve is sigmoidal, meaning that it has an inflection point at [*X*]=1/*K*_1_*K*_2_ (figure 2*c*). In the literature, as a first approximation, binding probabilities are often parametrized as Hill equations
2.11
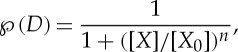

with *n* being the Hill coefficient (a measure of cooperativity) and [*X*_0_] being the half-saturation concentration. The hyperbola (2.6) (with *n*=1) and the sigmoid (2.10) (with *n*=2) can both be parametrized in this way (figure 2*c*). These parameters, among many others, are required to provide a detailed biochemical description of any genetic network.

## 3. Genetic networks

### (a) The network equation

Single genes are often regulated by multiple transcription factors that interact with one another. A classic example is the *lac* operon, which is regulated by both a repressor and an activator [20]. In eukaryotes, a single gene could be regulated by dozens of proteins. It is a remarkable fact that, using only thermodynamic constraints of the type we have considered, a promoter can be made to perform a variety of mathematical operations on its regulatory inputs. Specifically, the probability of occurrence of the transcriptionally active promoter configuration can be a complicated function of the concentration of various transcription factors [21–26]. These concentrations can themselves change over time owing to regulation of the genes encoding the transcription factors. If we wish to understand the behaviour of the system, we must therefore consider the regulatory network as a whole. We now try to arrive at a general mathematical description of such networks.

The rate of protein creation per promoter, *α*, is a product of the following terms: the probability that the promoter is transcriptionally active, the rate at which transcription proceeds irreversibly from the active state and the number of proteins translated per resulting transcript. Consider a cell that contains *n*_*P*_ copies of a gene encoding protein *Xi*. If the protein once created does not degrade, then the number of protein molecules *n*_*i*_ will obey
3.1
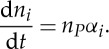

If the cell volume is *V* , then the protein concentration *x*_*i*_=[*Xi*] evolves as
3.2
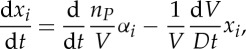

where the negative term arises owing to dilution.

Immediately after division, a bacterial cell contains a chromosome that has already begun to replicate. Depending on its position relative to the DNA replication origin, either one or two copies of each gene will be present at this stage. Every gene will be replicated once more before the cell is ready to divide again. The term *n*_*P*_/*V* can therefore vary by as much as a factor of two over the cell cycle. We will usually ignore this variation, assuming the promoter concentration to be constant, and absorbing it into the quantity *α*_*i*_. We will also assume that cell volume grows exponentially, so *V* (*t*)∝*e*^*γt*^. The growth rate *γ* is related to the cell doubling time *T*_D_ as 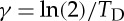. If the protein is subject to degradation in a first-order reaction, the rate constant of that reaction must be added to the dilution rate *γ* to give the net decay rate *γ*_*i*_. Protein degradation and dilution might themselves depend on the concentrations of some subset of proteins present in the system [27]. Finally, we have seen that the expression rate *α*_*i*_ can also depend on other protein concentrations. Taken together, these assumptions give
3.3
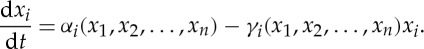

In many instances, network topology can be specified by sparse matrices of the form shown below, where only a few direct interactions generate non-zero matrix entries:
3.4
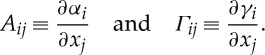

This apparently simple system of equations describes a typical genetic network. Of course, all the complex biochemistry is hidden within the functions *α*() and *γ*().

### (b) The network equation as an extension of Boolean threshold models

Equations of the general form (3.3) were first extensively studied by computational neuroscientists in their attempts to model neural networks [28]. In the neural context, the quantity *x*_*i*_ is the activity of a single neuron, and the function *α*() couples neurons to one another across synapses. The neural activity is a continuous variable, changing continuously over time, analogous to the expression level of a gene. Early models described neurons as binary units, which could perform thresholding operations (the so-called perceptrons [29]). In these models, *x*_*i*_ is 0 or 1, and neural activity is updated discretely according to the inputs received:
3.5
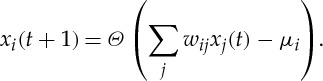

Here, *Θ*(*s*) is a step function, equal to 1 if *s*≥0, and 0 if *s*<0. The weight matrix *w*_*ij*_ describes the strength of the interaction between input neuron *j* and output neuron *i*. If the weighted input to neuron *i* crosses the threshold *μ*_*i*_, then the neuron is activated.

Starting with this binary description, we can generalize the model in many different ways. First, the synchronous update rule (‘=’) described earlier could be changed to an asynchronous update rule (‘:=’), selecting a random unit to update at each time step. Second, we could convert the binary activity variable to a continuous variable. In order to do this, we would need to select an appropriate function *α*() to describe how the neuron responds to its inputs. Typically, *α* is chosen to be a sigmoidal or threshold-like function, to which the step function is an approximation. This gives
3.6
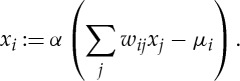

The dynamical variable is now continuous, but the model still operates in discrete time steps. Essentially, the neurons are assumed to adopt their new activities instantly upon update. Of course, the change of activity might occur gradually, with different neurons relaxing towards the steady state prescribed by (3.6) at different rates *γ*_*i*_:
3.7
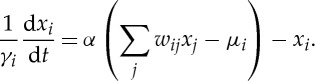

We thus arrive at an equation of the form (3.3). Note, however, that the function *α*() has a very special form, thresholding a weighted sum of inputs, an approximate phenomenological description of neural behaviour.

Moving back to genetic systems, how much can we learn by analogy with neural or electronic networks? It turns out that, when groups of genes are collected into a network, the resulting architecture is markedly different from that of the generic electronic circuit to which it is often compared. In the electronic case, large numbers of simple nodes are connected in complex ways. In the genetic case, the network is likely to be much more shallow, with each node, a promoter, executing more complex operations [14,21]. A single promoter is capable of responding in intricate ways to its inputs, and indeed, it is becoming clear that real single neurons might themselves be capable of sophisticated computations [30]. The simplicity and uniformity of electronic nodes have allowed us to model large electronic circuits very effectively. It is likely that there will never be an equivalent standard framework for the study of genetic systems—too much depends on the unique characteristics of each gene or protein. This is the biochemical complexity that makes the analysis of genetic networks challenging. Nevertheless, as we discuss in §4, topology proves to be a surprisingly useful determinant of network properties.

## 4. The emergent properties of networks

### (a) A biological wish-list

Imagine that we need to design a regulatory system to orchestrate one of the most intricate of all known biological processes, the development of a living embryo [31]. What are some of the tasks that need to be carried out, and some of the problems we might encounter along the way? We start with a fertilized egg that has undergone repeated divisions, thus producing a set of undifferentiated cells. Very soon, this embryo will begin to respond to maternal cues, in the form of spatial gradients of signalling molecules called morphogens, causing cells in different positions to express different sets of genes. Gene expression levels will need to vary significantly, as we move across segment boundaries: small changes in the levels of a signalling molecule must be amplified to produce large changes in expression. New transcription factors will be synthesized, triggering a subsequent round of gene expression. Cells will need to respond rapidly to these changes. At this stage, small errors in expression patterns must be avoided, as they would lead to larger and possibly lethal errors in downstream processes. The morphogen signals will eventually start to die away; the cells must nevertheless retain some memory of these signals, remaining firmly committed to their different fates. Developmental processes in different parts of the embryo will need to be synchronized: protein levels will need to oscillate periodically in time. And the list goes on.

**Figure 4.**
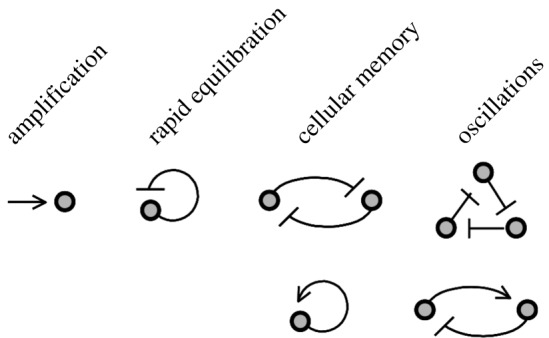
The emergent properties of networks: amplification by cooperative activation; rapid equilibration and noise reduction by negative feedback; memory and bistability by positive feedback and the flip–flop; oscillations by hysteretic and ring oscillators.

The surprising fact is, each of the tasks on our wish-list can be achieved by small networks of interacting genes (figure 4) [32,33]. In §4*b*, we survey a few simple networks that are able to generate, in principle, these various biologically desirable outcomes. Over the past decade systems such as those discussed here have been explored experimentally by synthetic biologists [34–36]: negative feedback for noise reduction [37,38]; positive feedback and the flip–flop for bistability [20,39–41]; and hysteretic and ring oscillators [26,42–44].

### (b) The dynamics of simple network topologies

**Figure 5.**
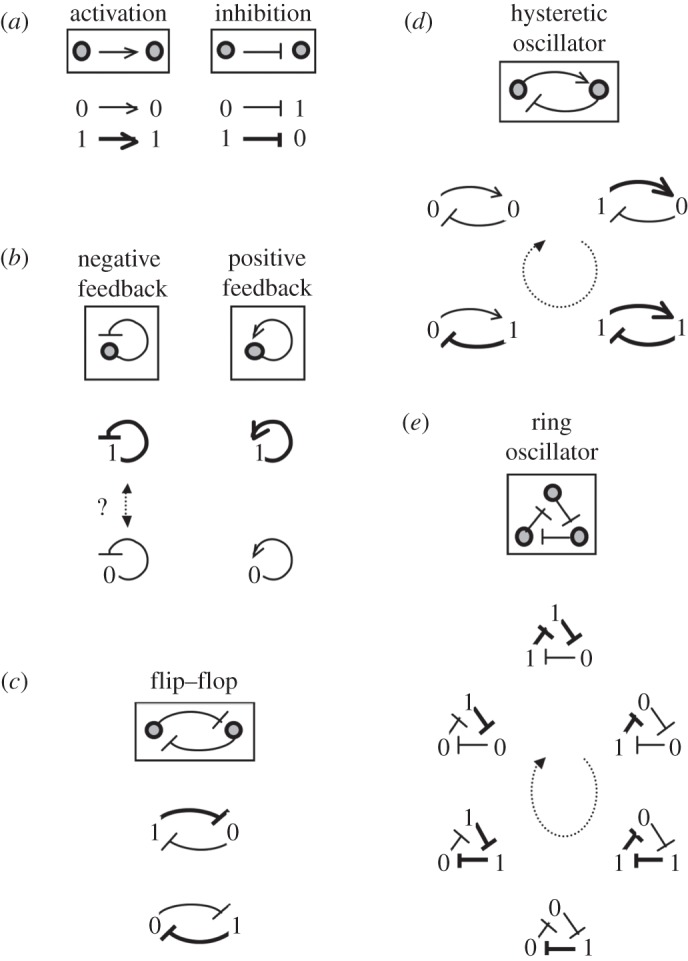
Simple binary networks. (*a*) Basic interactions between binary genes. Interactions are shown in bold if the regulator is active. (*b*) Feedback networks. The binary negative-feedback network does not have a self-consistent steady state. The binary positive-feedback network has two steady states, either active or inactive. (*c*) Flip–flop. If the first gene is active, then the second is inactive, and vice versa. As in the case of positive feedback, the system has two steady states. (*d*) Hysteretic oscillator. The dotted arrow represents transitions in time. The system cycles between states of high activator and high repressor expression. (*e*) Ring oscillator. The three genes cycle through high-expression states in succession.

Amplification by cooperative activation: consider a gene that encodes a protein *Y* and is regulated by an activator *X* (figure 5*a*). Cooperative interactions can result in a Hill-type dependence of the gene expression level on the activator concentration. Setting *x*=[*X*] and *y*=[*Y* ],
4.1
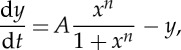

where for notational simplicity, *x* is measured in units of the half-saturation concentration (compare with (2.11)) and time is measured in units such that the decay rate of *y* is unity (compare with (3.3)). The value of the steady-state output, 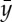, can depend sensitively on that of the input, 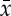:
4.2
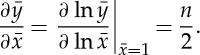

At high or low values of 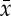, the value of 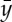 is close to either zero or *A* and is insensitive to changes in the input. However, near the threshold 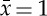, a certain fractional change in 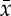 is amplified to produce an *n*/2 greater fractional change in 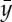: differential input signals will be amplified.

Rapid equilibration and noise reduction by negative feedback: consider what happens when a gene negatively regulates its own expression (figure 5*b*). Assume that the protein is a repressor that behaves as shown in (2.6):
4.3
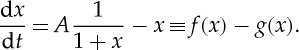

**Figure 6.**
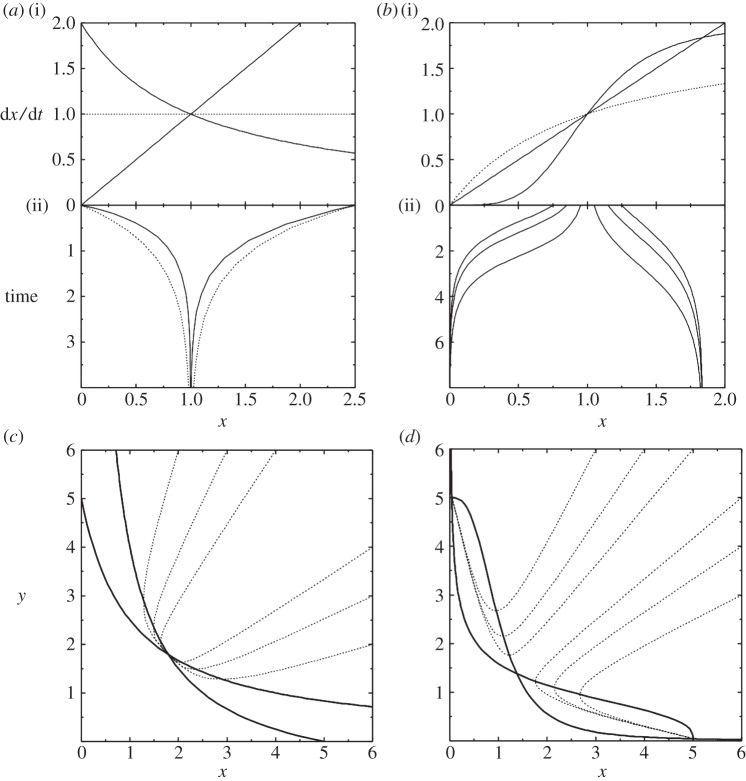
Continuous feedback networks. (*a*) Negative feedback. (i) Protein creation and degradation rates. (1) *f*(*x*)=2/(1+*x*) for the auto-repressed system. (2) *f*(*x*)=1 for the unregulated system. (3) *g*(*x*)=*x*. (ii) Solid lines show timecourses for the auto-repressed system; dashed lines show timecourses for the unregulated system. Negative feedback produces more rapid equilibration. (*b*) Positive feedback. (i) Protein creation and degradation rates. (1) For *f*(*x*)=2*x*/(1+*x*), the system has a single stable fixed point. (2) For *f*(*x*)=2*x*^4^/(1+*x*^4^), the system has two stable fixed points, separated by an unstable fixed point. (3) *g*(*x*)=*x*. (ii) Timecourses. Systems initialized at *x*>1 are driven to the high state, whereas those initialized at *x*<1 are driven to the low state. (*c*,*d*) Flip–flop. Graphs in *x*–*y* space show nullclines (solid) and trajectories (dashed) for equation (4.5) with *A*=5. (*c*) For *n*=1, the system has one stable state. (*d*) For *n*=4, the system has two stable states, one at high-*x* low-*y*, and the other at high-*y* low-*x*.

The steady state of the system corresponds to that concentration *x* at which the rate of creation *f*(*x*) and the rate of destruction *g*(*x*) balance one another. We see from figure 6*a* that the negative-feedback system settles into a steady state intermediate between 0 and *A* (something that cannot be captured in a pure binary description). If the expression level of the system is transiently increased above this steady state, the resulting drop in the creation rate quickly restores equilibrium. In fact, the auto-repressed system equilibrates more rapidly than an unregulated system with the same steady state, as shown in figure 6*a*; this has the effect of suppressing stochastic fluctuations [45].

Memory and bistability by positive feedback: we next allow the gene to positively regulate its own expression (figure 5*b*). This can be achieved by closing the loop in (4.1):
4.4
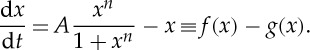

We see from the binary model that this system can have multiple steady states: a gene that is active will sustain its own expression, whereas one that is inactive will never become activated (figure 5*b*). In the continuous model, this would correspond to having multiple values of *x* at which the rates of creation and destruction balance one another. For hyperbolic activation (*n*=1), we find just one stable expression state. However, for sigmoidal activation (*n*>1), the system can have two stable states, separated by an unstable state that forms a threshold (figure 6*b*). Trajectories that begin above this threshold are driven to the high state, whereas those that begin below the threshold are driven to the low state. The behaviour of the system therefore depends on its history, a phenomenon known as hysteresis. Suppose that we begin with a group of cells in the low expression state, then fully induce expression in some of these cells by means of an external signal such as a morphogen. Even once this signal is removed, the induced cells will maintain their high-expression levels. The positive-feedback network thus forms the basis for cellular memory, allowing cells of identical genotype to achieve different phenotypes depending on the external signals received.

Memory and bistability with a flip–flop: a pair of genes that repress one another is similar to a single gene that activates itself (figure 5*c*). In the context of electronics, such systems are known as flip–flops. The binary version of this system is capable of maintaining two distinct internal states: if we choose one gene to be active, then the other must be inactive. In terms of concentrations,
4.5
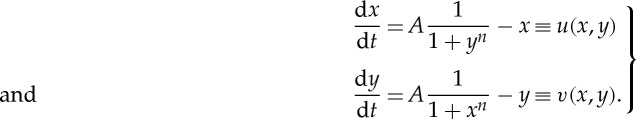

To understand system dynamics, it is useful to examine the curves *u*(*x*,*y*)=0, along which d*x*/d*t*=0, and *v*(*x*,*y*)=0, along which d*y*/d*t*=0. The fixed points or steady states of the system occur where these curves, known as nullclines, intersect. Once again, we must ask of each fixed point whether it is stable or unstable. In this case, a graphical analysis shows that, for *n*=1, the system has a single stable fixed point along the diagonal *x*=*y* (figure 6*c*). For *n*>1, this symmetric fixed point becomes unstable, and two asymmetric stable fixed points are created, one corresponding to high *x*-expression, and the other to high *y*-expression (figure 6*d*). As in the case of the positive feedback network, the flip–flop provides a mechanism for cellular memory.

Hysteretic oscillator: we again look at a system of two genes, but now one of them is an activator, while the other is a repressor (figure 5*d*). In a sense, this is an extended version of a negative feedback circuit we saw previously, and the binary model predicts that it should oscillate. Importantly, because the feedback now comes with a delay, oscillations can be shown to occur in the corresponding continuous system as well. Consider the following activator–repressor pair:
4.6
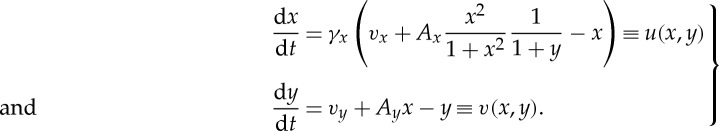

**Figure 7.**
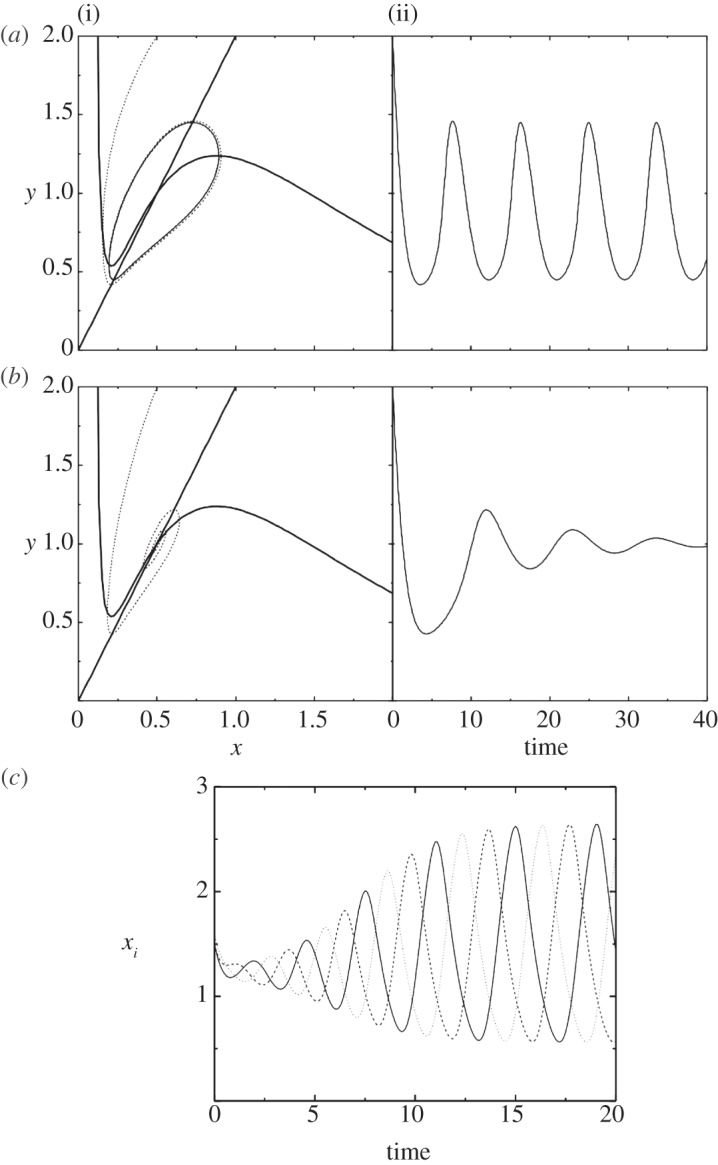
Continuous oscillators. (*a*,*b*) Hysteretic oscillator. We show results for equation (4.6), with *v*_*x*_=0.1, *v*_*y*_=0.0, *A*_*x*_=4.0, *A*_*y*_=2.0. (i) Shows nullclines (solid) and trajectories (dotted) in *x*–*y* space. (ii) Shows *y*(*t*). (*a*) For *γ*_*x*_=3.0, oscillations are damped and the system eventually reaches the fixed point. (*b*) For *γ*_*x*_=5.0, the fixed point is unstable, and the system enters a limit cycle oscillation. (*c*) Ring oscillator. We show results for equation (4.7), with *A*=4 and *n*=4. The graph shows the values of *x*_1_, *x*_2_ and *x*_3_ over time. The system eventually enters a limit cycle.

The nullclines intersect at a single fixed point, and the flows suggest oscillatory behaviour. If *x* is slow to respond to changes in *y*, this fixed point is stable and any oscillations are damped (figure 7*a*). However, if *x* responds sufficiently rapidly, the fixed point becomes unstable, and the system enters a sustained limit-cycle oscillation (figure 7*b*). Hysteretic oscillators of this kind are known to form the molecular basis for circadian rhythms and other types of periodic phenomena in living cells [46].

Ring oscillator: finally, let us consider a system with three genes, each repressing the next in sequence (figure 5*e*). The binary system is clearly oscillatory. The continuous analogue may be specified as
4.7
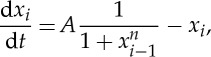

where *i*=0 is identified with *i*=3. The system has a symmetric fixed point *x*_*i*_=*x*_0_. For sufficiently high *n*, this fixed point can become unstable, forcing the system into a limit-cycle oscillation (figure 7*c*).

## 5. Separating biochemistry from topology

### (a) Estimating biochemical and topological complexity

Suppose we are given *N* distinct regulatable promoters, each of which has binding sites for up to *M* distinct transcription factors. In addition, we are given *N*_*ext*_ promoters whose transcriptional outputs can be controlled using extracellular signals. Each promoter can be made to express one or more transcription factors; the same transcription factor might be expressed by multiple promoters, in which case its total level is obtained by summing. We assume that the levels of all transcription factors can be measured. To simplify the discussion, we discretize the system so that all the inputs and outputs can take on any one of the states *x*∈{0,1,…,*Ω*−1} with inputs saturating at the maximal level. Reasonable values of these quantities are *N*, *N*_ext_ approximately 2–10, *M*∼2–5 [47], and *Ω*∼10.

A promoter is specified by defining its response to *Ω*^*M*^ distinct inputs. For each promoter *i*, let this information be summarized as a function *α*_*i*_(*x*_1_,*x*_2_,…,*x*_*n*_). The set {*α*_*i*_|*i*=1,…,*N*} represents the biochemical specification of the system. There are *Ω*^*Ω*^*NM*^^ possible biochemistries (though given the continuous and slowly varying nature of a promoter’s input–output function, the accessible biochemical space will in reality be much smaller than this).

We next turn to topology, which involves specifying which of the *N*+*N*_ext_ promoters is driving each of the *M* inputs of a given promoter. The *M*×(*N*+*N*_*ext*_) connectivity matrix for promoter *i* has the form
5.1
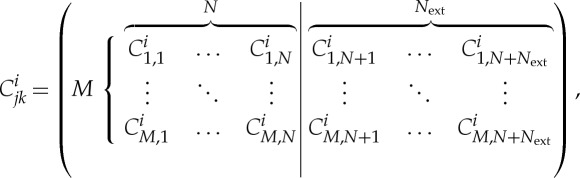

where the indices *j* and *k* run over inputs and promoters, respectively; and each entry can take on values 0 or 1. The set {*C*^*i*^|*i*=1,…,*N*} represents the topological specification of the system and there are approximately 2^*NM*(*N*+*N*_ext_)^ possible topologies (ignoring degeneracies). Notice that the biochemical space explodes much more rapidly than the topological space.

Consider a feedback network constructed with some complicated 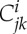. Such a network will have *N*^′^_*ext*_≤*N*_ext_ external inputs, and therefore can be put into *Ω*^*N*^′^_ext_^ configurations. How completely can we probe the biochemistry of such a system? To get a rough idea, let us make the following simplifying assumptions: for each external configuration, the feedback system achieves a unique steady state; and as we cycle through configurations, a given promoter cycles through a random sample (with repeats) of its *Ω*^*M*^ possible states. The probability that a given state is missed over *Ω*^*N*^′^_ext_^ samples is 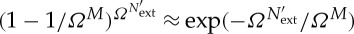. Therefore, the expected number of distinct states sampled by each promoter is 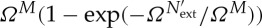. The depth of biochemical characterization is essentially a step function: if *N*^′^_ext_<*M* our sampling is extremely sparse; if 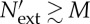 we hit nearly all possible states; and with *Ω*^*M*^ samples our fractional coverage is (1−1/e).

If *N*_ext_≥*M*, we can choose to *construct* a synthetic genetic network with the trivial feed-forward architecture (as reported in Rai *et al.* [26]):
5.2
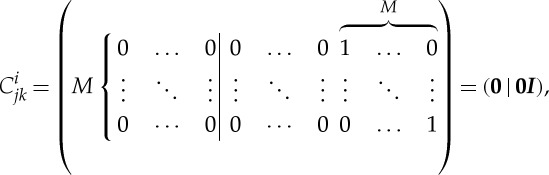

where **0** is the zero matrix and ***I*** is the identity matrix. This allows us to perform a complete biochemical characterization, in which we determine all the functions *α*_*i*_, using exactly *Ω*^*M*^ external configurations. Having done the feed-forward characterization we can, in principle, predict the response of any other topology under all of its *Ω*^*N*′_ext_^ external configurations. An experimental demonstration of this feed-forward-to-feedback predictive procedure was reported by Rai *et al.* [26]. For *N*′_ext_>*M*, this type of prediction is clearly efficient: a large number of feedback responses can be predicted from a relatively small number of feed-forward measurements. However, in practice, it is often the case that even *Ω*^*M*^ is large in absolute terms, making a complete biochemical characterization unfeasible.

### (b) Case study: bacterial cell-to-cell communication

**Figure 8.**
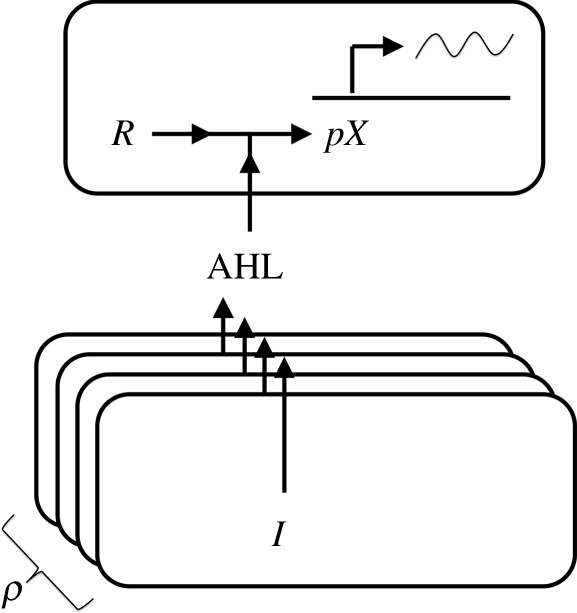
Schematic of an *I*/*R* quorum-sensing system. Cells have number density *ρ*. The intracellular enzyme *I* synthesizes the chemical signal AHL, which diffuses into the medium and subsequently into other cells. The transcription factor *R*, when bound to AHL, activates transcription of mRNA at the promoter *pX*. For clarity, we have separated the ‘issuing’ and ‘receiving’ of the chemical signal, but these processes happen simultaneously within each cell.

There are several natural contexts in which bacterial cells in a population stand to benefit by coordinating their actions [48]. Many bacterial species achieve such coordination through chemical communication channels that work on the following principle [49]. Any cell in the population can ‘issue’ a signal using an enzyme designated *I*; this enzyme generates a molecule known as acyl-homoserine lactone (AHL) that can diffuse freely between cells. Cells ‘receive’ this signal using a transcription factor designated *R*; when *R* is bound to AHL it functions as an activator, driving transcription at a promoter henceforth designated *pX*. The capability of *I*/*R* systems to issue and receive signals can have a variety of uses [50]. Because the concentration of AHL in the medium is a readout of the density of cells issuing the signal, one hypothesis is that these systems allow cells to tune their transcriptional response as a function of population density (figure 8)—hence the term ‘quorum sensing’. For example, cells infecting a host can remain quiescent until they reach a critical density, staying hidden from the host’s immune system until they are ready to launch a virulent attack [51]. Topologically, *I*/*R* quorum-sensing systems are interesting because they are invariably found in a particular positive-feedback configuration: the enzyme *I* is expressed downstream of the *R*-dependent promoter *pX* [26,52].

A computational and experimental characterization of *I*/*R* systems has been reported previously [26]. We revisit those results in the context of the biochemical and topological framework developed here. The key variables are (figure 8): the bacterial cell density *ρ*; the concentration *ϕ* of AHL in the medium; and the intracellular concentrations *Y*_*I*_ and *Y*_*R*_ of the enzyme *I* and transcription factor *R*. AHL levels will be proportional both to the enzyme levels and to cell density: *ϕ*(*t*)=*μρ*(*t*)*Y*_*I*_(*t*). The transcriptional output of promoter *pX* is a function of instantaneous AHL and *R* levels. This biochemistry is summarized:
5.3
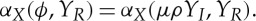

Given two external promoters *pA* and *pB*, the system can be wired into the following topologies:
5.4
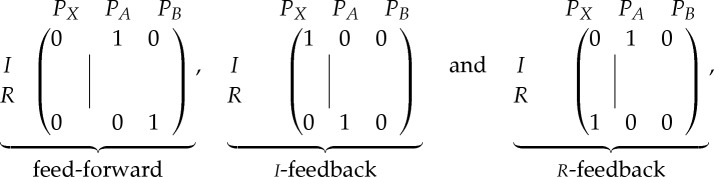

where matrices of the format (5.1) specify which promoters are driving which of the two inputs of promoter *pX*. If the proteins *I* and *R* have translation rates *Q*_*I*_, *Q*_*R*_ and decay rates *γ*_*I*_, *γ*_*R*_, respectively, the feedback systems are described by the following differential equations:
5.5
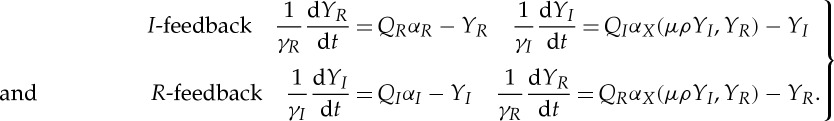

**Figure 9.**
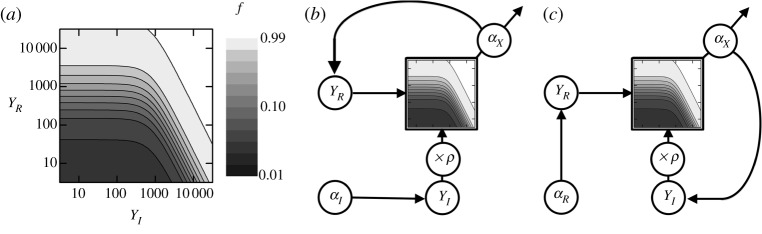
*I*/*R* feedback systems. (*a*) The input–output function of *pX*: the output transcription rate as a function of *Y*_*I*_ and *Y*_*R*_ at a fixed cell density *ρ*. The contour plot shows the value of *α*_*X*_(*μρY*_*I*_,*Y*_*R*_), as measured in Rai *et al*. [26]. (*b*,*c*) Feedback topologies. Either *R* or *I* is controlled externally, while the other protein is expressed from the promoter *pX* with transcription rate *α*_*X*_(*μρY*_*I*_,*Y*_*R*_). The same promoter can also drive further outputs. The two topologies are different because the function *α*_*X*_() is asymmetric, and because it is only the term *Y*_*I*_ that is multiplied by the cell density *ρ*. (*b*) *R*-feedback. (*c*) *I*-feedback.

Here, *α*_*I*_ and *α*_*R*_ are control parameters: transcription rates that are constant in time but whose values can depend on external inputs; the function *α*_*X*_() embodies the frozen biochemical parameters; and the structure of the equations indicates the feedback topology. There are evidently two reasons why the responses of *R*-feedback and *I*-feedback systems might differ. The first is biochemical: the promoter logic *α*_*X*_(*μρY*_*I*_,*Y*_*R*_) is an asymmetric function of its two inputs *Y*_*I*_ and *Y*_*R*_ (figure 9*a*). The second is structural or topological: the input *Y*_*I*_ is multiplied by the cell density, whereas the input *Y*_*R*_ is fed in directly (figure 9*b*,*c*) causing these two variables to influence the dynamics in completely distinct ways.

**Figure 10.**
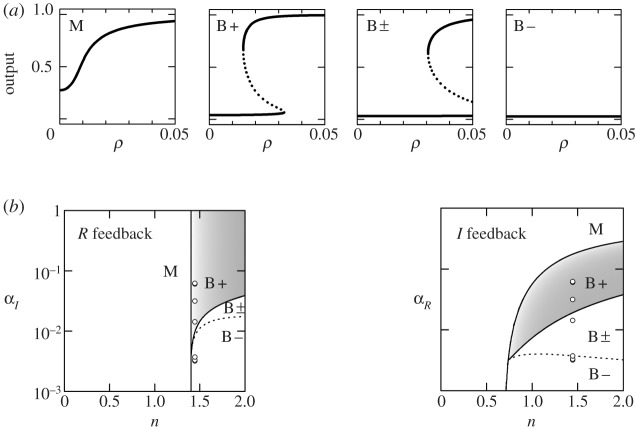
Density-dependent responses. (*a*) Four types of responses: (M) monostable, where transcription smoothly increases with cell density; (B+) bistable, with a threshold density at which transcription abruptly increases; (B±) bistable and hysteretic at the terminal density, where high and low transcription states coexist; (B−) bistable but uninduced even at the terminal density, since the potentially bistable region is never reached. (*b*) Regions of {*α*,*n*} space that generate each response type; *α* represents the external control parameter, whereas *n* represents the Hill coefficient based on a parametrization of the input–output function *α*_*X*_(*μρY*_*I*_, *Y*_*R*_) [26].

If cell density varies slowly compared with intracellular protein concentrations, equation (5.5) can be solved to obtain quasi-steady-state values *Y*_*I*_ and *Y*_*R*_ as functions of *ρ*. Under positive feedback, two distinct classes of responses can arise (figure 10*a*). For monostable responses (type M; mnemonic sMooth), transcription increases smoothly with cell density. For bistable responses (type B; mnemonic aBrupt), there is a range of cell densities over which two stable transcription levels coexist. For each topology, a bifurcation analysis can be used to obtain regions of parameter space that give rise to the different response types [26], supporting information. Figure 10*b* shows a two-dimensional slice of the parameter space: a biochemical parameter *n* (the Hill coefficient of *R*-DNA binding, which plays a key role in determining the form of *α*_*X*_()) is varied along the *x*-axis; the control parameters *α*_*I*_ or *α*_*R*_ are varied along the *y*-axis. We see that the *R*-feedback topology is constrained: it is restricted to a single response type independent of the regulator level, once biochemical parameters are frozen. However, the *I*-feedback topology is versatile: it can be tuned between smooth and abrupt density-dependent response types by varying the regulator alone. This versatility might underlie the observed preference for *I*-feedback systems among diverse bacterial species: an organism that is able to rapidly modify its response in the face of an uncertain and fluctuating environment gains a crucial fitness advantage. Versatility is a purely topological property of the system, made without reference to specific biochemical parameter values.

## 6. Conclusion

**Figure 11.**
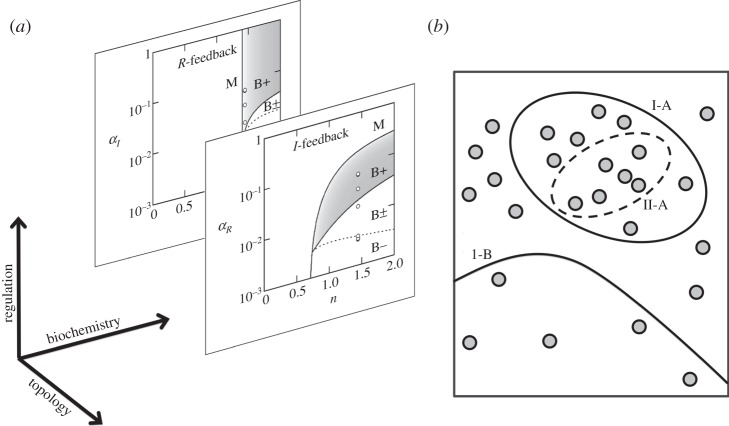
Using topology to tame biochemistry. (*a*) Regulation, biochemistry and topology can each be used to modulate the response of a genetic network, but on successively longer time scales. (*b*) We show a slice of biochemical space in which two network topologies (I and II) can potentially generate two different types of responses (A and B) within the regions indicated. Grey dots represent the unknown, *a priori* distribution of parameter values. Although region I-B appears larger than region I-A, topology-I is much more likely to generate type A responses compared with type B responses because of the increased density of dots in region I-A. However, because region I-A completely contains region II-A, we can say that topology-I is more likely to generate type A responses than topology-II is, regardless of the density of dots.

There are three types of changes that can be used to modulate the response of genetic networks, operating on completely distinct time scales (figure 11*a*). Control parameters (such as the transcription rates *α*_*I*_ or *α*_*R*_) are the *software*: they can respond directly and dynamically to external inputs, and vary on time scales from minutes to hours. Biochemical parameters (such as the Hill coefficient *n*) are the *firmware*: they can be changed incrementally by mutations, infrequent events that might become fixed in a population only over hundreds of generations. Network topology is the *hardware*: it is possible to switch topology but this requires rare, potentially disruptive, large-scale DNA rearrangements. The topological hardware and biochemical firmware are essentially frozen, leaving only the regulated software to vary freely at short time scales.

When studying natural genetic networks, the approach to take depends on the extent of available data. If topology is known and key parameters identified, we can use experimental measurements to constrain as many parameters as feasible. Of the remaining parameters we can try to identify a few that are expected to be critical, and investigate all possible system behaviours as their values are varied. This approach is incomplete, however, because of a further unknown that is often ignored: we rarely, if ever, know the *a priori* distribution of parameter values that are likely to occur in nature. It is therefore impossible to estimate or compare the volumes of regions in parameter space that give rise to any set of specified behaviours (such as A or B; figure 11*b*). Even in this situation, topology provides a useful organizing framework. Consider the region of parameter space of some genetic network associated with some desired behaviour. If this region in the case of topology-II is completely contained within that in the case of topology-I, then we can be certain that the topology-I is more likely to generate the desired behaviour, without knowing anything about the likelihood of occurrence of parameters (figure 11*b*). The analysis thus generates a partial ordering among topologies independent of the actual biochemistry, and suggests a means to search the space of all possible topologies for interesting networks. Searching through topologies in this manner might be the only approach possible if the very existence of certain interactions is in doubt. For each topology, we would scan over parameter values to identify the range of possible behaviours. It could be the case that several topologies are consistent with some desired outcomes. In that case, it might be necessary to add additional biologically relevant constraints: robustness to parameter variation; adaptation to external changes; power consumption efficiency; and so on. The approach of searching topological space with constraints is emerging as a powerful means to understand the design principles of complex genetic networks in the absence of detailed biochemical data [53–58].

We might soon achieve a nearly complete understanding of certain simple organisms through a systematic analysis of the networks that govern their behaviour. Eventually such techniques might even give us predictive power, allowing us to guess at the inner workings of organisms based solely on the annotated sequences of their genomes. However, on very long time scales, the structure of a network must itself be dynamic: natural selection can be thought of as driving a search through topological space, converging on network architectures that generate biologically useful outcomes [59,60]. As more and more genome sequences enter the databases, we can begin to catalogue regularities in network architecture, or striking differences between different species. Once enough such patterns are known, it might be possible to shift our focus away from the question that concerned us here, of what genetic networks do, towards the broader question of how such networks came to be.
